# Clinical Safety of Pudilan Xiaoyan Oral Liquid for the Treatment of Upper Respiratory Tract Infection in the Real World: Protocol for a Prospective, Observational, Registry Study

**DOI:** 10.2196/65789

**Published:** 2025-03-21

**Authors:** Mengmeng Wang, Lianxin Wang, Fumei Liu, Renbo Chen, Zhifei Wang, Xin Cui, Yuanyuan Li, Yanming Xie

**Affiliations:** 1 Institute of Basic Research in Clinical Medicine China Academy of Chinese Medical Sciences Beijing China

**Keywords:** Pudilan Xiaoyan oral liquid, PDL, upper respiratory tract infection, URTI, registry, adverse drug reaction

## Abstract

**Background:**

*Pudilan Xiaoyan* oral liquid (PDL) is a proprietary Chinese medicine preparation widely used for upper respiratory tract infection, known for its significant therapeutic effects. However, the safety profiles reported in several observational studies vary, and these studies primarily focus on efficacy rather than specifically addressing safety concerns, thus representing inadequate safety monitoring.

**Objective:**

This study aimed to investigate the incidence of adverse drug reactions (ADRs) associated with PDL and explore the factors contributing to these reactions.

**Methods:**

The study is a prospective, observational, multicenter, hospital-based surveillance study. A total of 17 hospitals from China are involved. The study is expected to enroll a large sample of 10,000 patients aged between 18 and 80 years with upper respiratory tract infection who were prescribed PDL. The patients’ data, including demographics, medical history, diagnostic information, medication details, adverse events, and laboratory test results, will be monitored. The occurrence of ADRs will be recorded. The primary outcome is the incidence of ADR. Secondary outcomes are the ratio of patients whose body temperature return to the normal range (cases of body temperature normalization and the duration for achieving normal body temperature within a 3-day period will be documented) and changes in liver and kidney function (occurrence of drug-induced liver injury and acute kidney injury). Descriptive analyses will be performed for the primary and secondary outcomes. A cohort, nested, case-control study design will be used. If one patient has an ADR, then 4 patients without ADRs will be matched as the control group according to gender, age within 5 years, drug batch, and other factors, at a ratio of 1∶4 to compare the symptoms related to ADRs. The differences of ADR incidence among the possible influencing factors will be compared separately to find the factors with large differences. Then, synthetic minority oversampling technique and group least absolute shrinkage and selection operator methods will be used to identify factors influencing the occurrence of ADRs. Finally, propensity scoring methods will be used to control for confounding variables. The progress of each subcenter will be closely monitored, and the incidence of ADR will be systematically calculated. Furthermore, the characteristics and influencing factors of ADR will be analyzed, along with an investigation into its geographical distribution.

**Results:**

The study began on July 17, 2019. Due to the limited number of eligible patients, missed follow-ups, and the huge clinical burden caused by public health events in 2019, the final case will be enrolled on August 30, 2025.

**Conclusions:**

This study will obtain safety results of PDL in the real world and provide guidance on the clinical safety of traditional Chinese medicine formulations.

**Trial Registration:**

ClinicalTrials.gov NCT04031651; https://clinicaltrials.gov/study/NCT04031651

**International Registered Report Identifier (IRRID):**

DERR1-10.2196/65789

## Introduction

Upper respiratory tract infection (URTI) involves inflammation of the respiratory mucosa from the nose to the lower respiratory tract; it causes localized symptoms that constitute several overlapping syndromes: pharyngitis, common cold, sinusitis, and bronchitis [1]. Bacterial or viral infections may be the cause of URTI, but antibiotics are recommended only for the former [1]. It is a self-limiting viral infection [2]. Currently, considering the treatment of symptoms for URTI and anti-infective therapy [3], the efficacy remains unsatisfactory with notable adverse reactions [4]. *Pudilan Xiaoyan* oral liquid (PDL) is a Chinese patent medicine, collected in the *Chinese Pharmacopoeia*. It has been widely used for URTI and has obtained good curative effect [5,6]. It contains drugs such as *Scutellaria baicalensis*, *Corydalis bungeana*, *Taraxacum mongolicum*, and *Isatis indigotica* [7]. All four herbs have the traditional Chinese medicine effects of removing heat and toxic materials, cooling blood, and reducing swelling. Besides, they also have anti-inflammatory, analgesic, antibacterial, and other biological effects [8]. Despite the efficacy of PDL, it is also necessary to monitor its safety.

In terms of safety, among the biological mechanisms of PDL, *S baicalensis*, *C bungeana*, *T mongolicum*, and *I indigotica* have been identified as herbs with low toxicity. The aforementioned drugs, however effective, are associated with gastrointestinal adverse reactions due to their bitter cold properties. Additionally, *S baicalensis* exhibits limited embryotoxicity and hepatotoxicity and can induce allergic reactions leading to vesicular eruptions [9]. *T mongolicum* primarily contributes to reversible toxicity targeting red blood cells [10], while *I indigotica* possesses nephrotoxic properties and may impose a burden on the urinary system with prolonged use [11]. The formulation of *C bungeana* contains certain hepatotoxic effects [12]. Therefore, it is necessary to investigate the safety of PDL since it is composed of the aforementioned herbal groups.

Monitoring the unintended effects of a drug (pharmacovigilance or drug safety) is crucial due to limited knowledge of rare or late-stage side effects upon market release of new drugs [13,14]. In recent years, numerous related studies have reported the clinical efficacy of PDL; however, there were few independent safety studies on it, most of which were small scale and exhibited significant variations in reported safety outcomes across different studies [15-18], resulting in inadequate understanding of its safety monitoring. In summary, published safety data on adverse effects of PDL therapy were insufficient, thereby impeding the use of standardized recommendations that are necessary for ensuring drug safety.

Although Chinese patent medicine generally exhibits a low incidence of adverse reactions, the evidence pyramid classification derived from effectiveness evaluations primarily considers randomized controlled trials as high-level evidence. However, this classification standard may not fully meet the evidence requirements for postmarketing drug safety evaluation due to limitations such as short intervention courses or small sample sizes, often resulting in an inability to detect ADRs and differences between groups. For the above reasons, this study intends to adopt a large-scale, multicenter, long-term, noninterventional, observational study design to comprehensively analyze and report on the occurrence of adverse events (AEs) [19].

The mechanism of kidney metabolism and excretion of various drugs and toxins is an important cause of drug nephrotoxicity [20], while studies have shown that most of the AEs related to the urinary system were from treatment with PDL [21]. It can be seen that the kidney damage caused by PDL needs further investigation. As hepatic function plays an essential role in drug metabolism [22], clinical studies are necessary to determine the characteristics of PDL-related hepatotoxicity. Additionally, the influencing factors of other PDL-related adverse reactions are still unclear and may be related to the inherent characteristics of the drug itself, improper usage and dosage, improper combination, repeated administration, or use without indications, which need further investigation and study. Furthermore, a comprehensive study regarding the adverse effects associated with PDL use in patients is imperative to optimize the risk-benefit ratio and alleviate strain on limited health care resources.

## Methods

### Research Design

**Figure 1:**
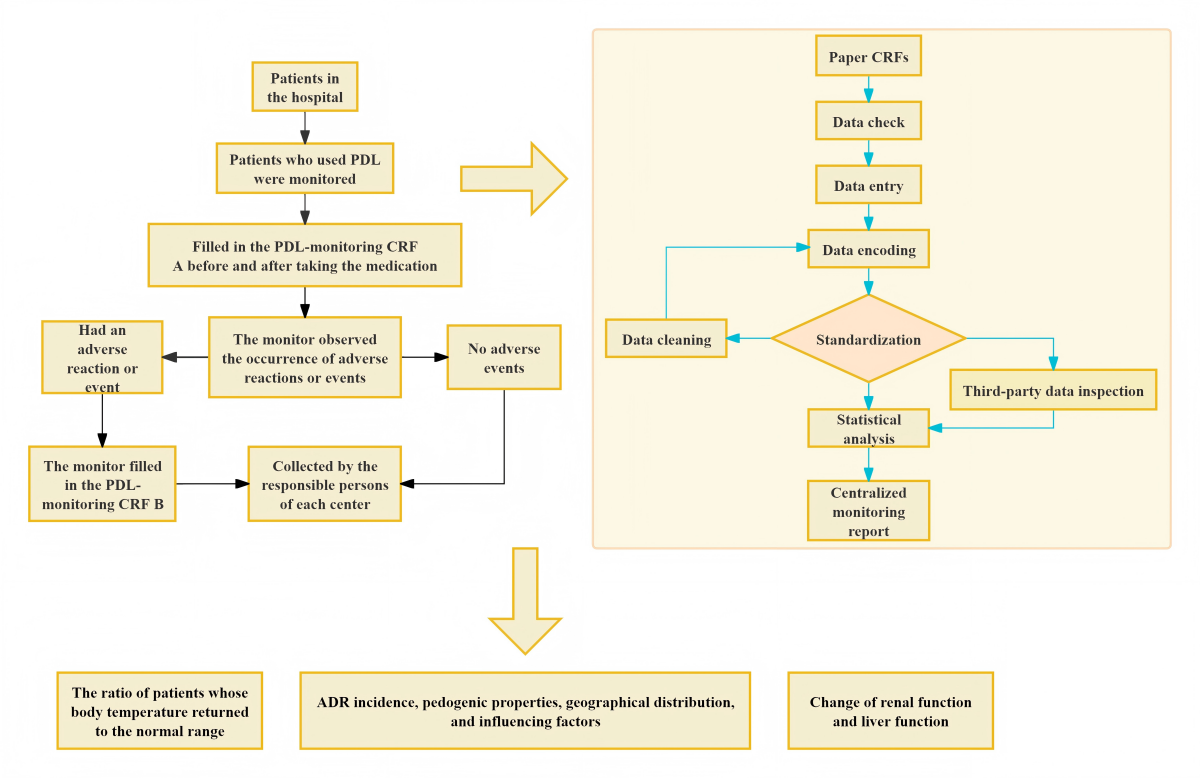
Study flowchart. ADR: adverse drug reaction; CRF:case report form; PDL: Pudilan xiaoyan oral liquid.

This is a prospective, observational, multicenter, hospital-intensive monitoring, and continuous registry study. We used the SPIRIT (Standard Protocol Items: Recommendations for Interventional Trials) checklist when writing our report [23]. The study flowchart is shown in Figure 1.

### Organization

**Figure 2:**
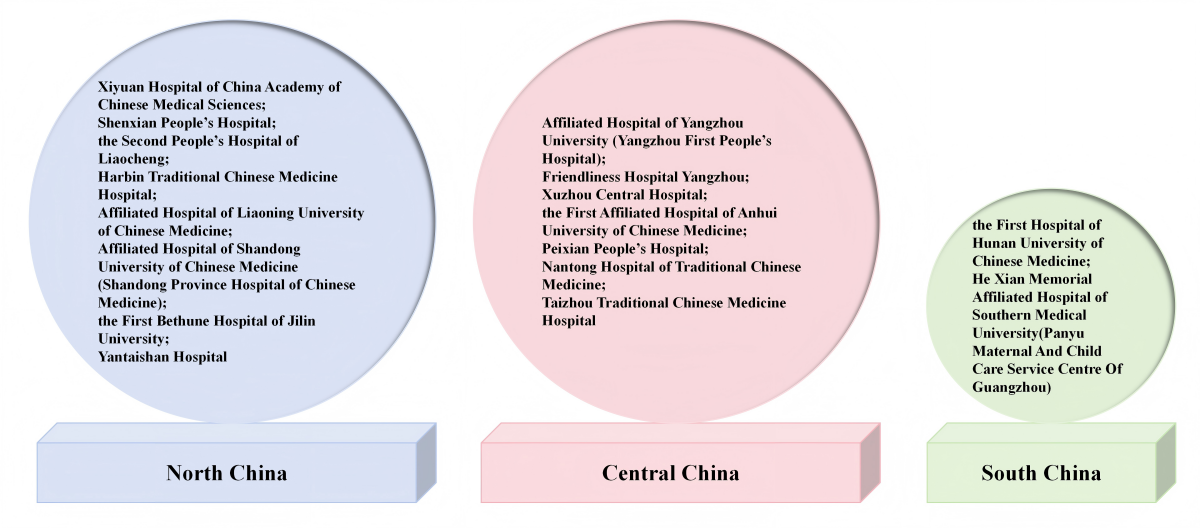
The geographical location of the 17 hospitals in China involved in this study.Light blue represents North China, light pink represents Central China and light green represents South China.

A total of 17 hospitals in China are involved in this study: Xiyuan Hospital of China Academy of Chinese Medical Sciences, the First Hospital of Hunan University of Chinese Medicine, Nantong Hospital of Traditional Chinese Medicine, Shenxian People’s Hospital, the Second People’s Hospital of Liaocheng, He Xian Memorial Affiliated Hospital of Southern Medical University(Panyu Maternal And Child Care Service Centre Of Guangzhou), Harbin Traditional Chinese Medicine Hospital, Affiliated Hospital of Yangzhou University (Yangzhou First People’s Hospital), Friendliness Hospital Yangzhou, Xuzhou Central Hospital, Peixian People’s Hospital, Affiliated Hospital of Liaoning University of Chinese Medicine, Affiliated Hospital of Shandong University of Chinese Medicine (Shandong Province Hospital of Chinese Medicine), the First Bethune Hospital of Jilin University, the First Affiliated Hospital of Anhui University of Chinese Medicine, Yantaishan Hospital, and Taizhou Traditional Chinese Medicine Hospital. Figure 2 shows their specific locations.

### Sample Size

In accordance with the requirements of the *Guide to Key Drug Monitoring for Manufacturing Enterprises* [24], and referring to the 2013 *Technical Specifications for Intensive Hospital Safety Monitoring of Post-Marketing Chinese Medicine* [25], the “rule of threes” was used for sample size calculation, which states that “if no specific event is observed in X existing cases, there is a 95% likelihood that the occurrence rate of such an event is ≤3/X.” Based on the detection rate of rare ADRs (1/10,000 to 1/1000), it is estimated that 3000 cases would need to be collected. However, due to the low probability of ADR occurrences investigated in this study, actual monitoring will be extended to 10,000 cases.

### Monitoring Time and Follow-Up

Patients are monitored throughout the medication period in the hospital. The treatment duration of PDL for URTI ranges from 3 to 14 days. This study will include a follow-up period of 7 days after the completion of observation treatment [26], aiming to assess the drug’s long-term efficacy and observe any delayed ADRs. The follow-up will be conducted either through face-to-face meetings or via telephone communication. We will use systematic methods and reminders to schedule appointments with patients.

### Criteria

The inclusion and exclusion criteria are reported in Textbox 1.

**Inclusion and exclusion criteria.**

**Inclusion criteria**

- Patients with upper respiratory tract infection (URTI) who are administered
  Pudilan Xiaoyan
  oral liquid; the diagnostic criteria for URTI are based on the disease code J06.900 of the
  International Classification of Diseases, 11th Revision
- Aged between 18 and 80 years

**Exclusion criteria**

- Lactating women, pregnant women, those planning to become pregnant, patients with psychiatric conditions, and others

### Instructions for Taking Medicine

In the respiratory and emergency departments of the hospitals under surveillance, the medication of PDL is observed for those aged 18-80 years who strictly follow the prescribing instructions: taking PDL 10 mL at a time, 3 times a day. During hospitalization, the patient must take PDL at least once. The patients will be visited and checked before and after taking the medicine.

### Monitoring Content and Data Administration

#### Monitoring Patient Data, Demographic Information, Diagnosis, Complications, and Medication Information

A collaborative monitoring approach involving doctors, nurses, and pharmacists will be used in this study. They will receive relevant training in surveillance knowledge.

#### Basic Information Form for Administering PDL

The researchers will record the AE information collected during the study period into the safety monitoring case report form (CRF). All patients will be required to complete CRF A (PDL basic information form). If the monitored patient experiences an ADR or AE during the medication period, the monitor will need to fill out CRF B. We will check the monitoring data; enter, encode, standardize, and clean the data; and then conduct a third-party data inspection. Any discrepancies in the data will be addressed by two data administrators who will resolve them after independently verifying the original CRFs.

### Interpretation of ADR Causality

In this study, AEs, ADRs, and serious ADRs will be judged according to the *Administrative Measures on Reporting and Monitoring for Adverse Drug Reaction* issued by the Ministry of Health in 2011 [27]. The evaluation of ADRs follows a 3-level decision-making process, comprising initial reporting by clinical practitioners, assessment by the Expert Committee for ADR Monitoring at the surveillance center, and final determination by panel of senior domestic experts. Based on established assessment criteria, the causal relationship between AEs and PDL will be evaluated according to five key parameters: (1) temporal plausibility, (2) consistency with known mechanisms of action or established adverse reaction profiles, (3) positive dechallenge response, (4) positive rechallenge response, and (5) exclusion of alternative explanations. The correlation between AE and PDL will be divided into six grades: positive, probable, possible, possibly unrelated, to be evaluated, and unable to be evaluated. Those judged to be the first three grades will be classified as ADRs, those judged to be “possibly unrelated” will be classified as AEs, and those judged as “to be evaluated” or “unable to be evaluated” should be rejudged in combination with the original monitoring data. We will conduct thorough evaluations to ultimately confirm the incidence of ADRs associated with PDL. This approach helps to minimize potential biases associated with patient self-reporting and variations in medical practices across different centers. The occurrence characteristics and processes of ADRs will be meticulously documented, with particular emphasis on the timing of ADR onset, the implementation time of intervention measures for ADRs, and the termination time of ADRs.

### Quality Control of ADRs

Each monitoring subcenter will formulate the first-level, quality-control rules and quality-control list according to the content of the monitoring program. The information system of each monitoring unit will be reviewed to ensure that the clinical study data are authentic and traceable. The implementation of the study will adhere to the research protocol and standard operating procedures in order to ensure the authenticity and reliability of the research data. The data collected are presented in Table 1.

**Table 1:** Basic information form for administering Pudilan Xiaoyan oral liquid^a^.

Basic information	Before taking the medicine	After taking the medicine		
		7 days	14 days	21 days
Age	✓			
Sex	✓			
Smoking history	✓			
Occupation	✓			
Expense category^b^	✓			
Pathways to admission	✓			
Drinking alcohol	✓			
Western medicine diagnosis	✓			
Traditional Chinese medicine diagnosis	✓			
Past medical history	✓			
Personal history of allergies	✓			
Allergic manifestation	✓			
Previous other adverse drug reactions or events	✓			
Main drug combination: Western medicine, antibiotics, antipyretic analgesia, proprietary Chinese medicine, or other drugs		✓	✓	✓
Blood routine before and after treatment, white blood cell count, granulocyte, routine urine examination, liver function, renal function, ECG^c^ (test sheet within 30 days before treatment), C-reaction protein, influenza virus detection, pathogen detection, and other tests	✓	✓	✓	✓
Adverse reaction occurrence (yes or no)		✓	✓	✓

^a^Patients are visited and examined before and after taking the medication.

^b^Expense category refers to the classification of expenses, such as out-of-pocket (self-funded) costs, expenses covered by medical insurance, and other payment types. It helps in understanding how different types of expenses are categorized based on their funding source or payment method.

^c^ECG: electrocardiogram.

### Outcome Measures

#### Primary Outcome Measure: Incidence of ADRs

The incidence of ADRs is the primary outcome measure, and we will identify factors that contributed to the occurrence of ADRs. The incidence of ADR will be primarily categorized in accordance with the criteria proposed by the Committee of International Organization of Medical Sciences: very common (≥10%), common (1%-10%, including 1%), occasional (0.1%-1%, including 0.1%), rare (0.01%-0.1%, including 0.01%), and very rare (<0.01%) [28], as well as according to the classification of ADR symptoms by the World Health Organization (WHO) [29]. The WHO classification of organs and systems involved in ADRs will also be referenced [30]. The incidence characteristics and regional distribution will be subsequently analyzed, while concurrently investigating the influencing factors (Table 2).

**Table 2:** Centralized monitoring results of Pudilan Xiaoyan oral liquid in the registered hospitals.

Monitoring result	Description
Categorization	The ADR^a^ was categorized based on general, novel, and severe classifications The classification was based on the organs and systems affected by the ADR The symptoms of the ADR were classified
ADR incidence	The specific event features of each ADR included the incidence rates of various categories and whether they are infrequent or exceptionally rare
Pedogenic properties	Occurrence characteristics of ADRs specific to each category
Geographical distribution	The distribution of ADRs is primarily observed in North China, Central China, South China Each region exhibits distinct characteristics of ADRs
Influencing factors	Days of medication, age, drug combination, etc

^a^ADR: adverse drug reaction.

#### Secondary Outcome Measures

##### The Ratio of Patients Whose Body Temperature Returned to the Normal Range

The cases of body temperature normalization and the duration for achieving normal body temperature within a 3-day period will be documented. The body temperature will be recorded prior to medication, on the first day after medication, and on the second and third days after medication. Daily measurements will be conducted by professional nurses using mercury thermometers. The time of normal body temperature restoration is defined as when the body temperature drops below 37.3 °C after the initial medication administration, with no recurrence observed within a 24-hour period. Thus, the ratio of normalization represented the percentage of cases in which individuals return to a normal body temperature within the total number of cases in the group.

##### Change in Liver Function

We will observe liver function in at least 3000 patients before and after medication. Liver function indexes will include alanine transaminase (ALT) and aspartate transaminase. To observe whether the above liver function indexes appear abnormal after medication, we will use the 2011 International Association for Severe Adverse Reactions–recommended biochemical diagnostic criteria for drug-induced liver injury [31]. Drug-induced liver injury is defined as meeting one of the following conditions:

1. ALT ≥5× upper limit of normal (ULN);
2. Alkaline phosphatase ≥2×ULN, especially with 5’-nucleotidase or γ-glutamyl transpeptidase elevation, excluding alkaline phosphatase elevation caused by other diseases; or
3. ALT ≥3×ULN and total bilirubin ≥2×ULN.

##### Change in Renal Function

We will observation renal function in at least 3000 patients before and after medication. The renal function indexes will include creatinine and blood urea nitrogen. To observe whether the above renal function indexes appear abnormal after medication, the diagnostic criteria for acute kidney injury will be defined based on the Kidney Disease: Improving Global Outcomes serum creatinine criteria [32] (at least 50% increase in creatinine within 7 days or a 26.5 μmol/L increase within 48 h).

The time frame for all four result items is 7 days (Table 2).

### Statistical Analysis

Data review and cleaning work will take the paper monitoring form as the original record, delete redundant and repetitive parts, supplement missing data, modify wrong values, and report about abnormal values. The basic characteristics of the patients will be included. Descriptive analyses will be performed for the primary and secondary outcomes. SPSS Statistics 26 software (IBM Corp) will be used for descriptive analysis. Data conforming to a normal distribution will be described by means and SDs, and those conforming to a skewed distribution will be described by median, minimum, maximum, and IQR scores. Categorical data will be described by frequencies and percentages. A cohort, nested, case-control study will be used. If 1 patient has an ADR, then 4 patients without ADRs will be matched as the control group according to gender, age with 5 years, drug batch, and other factors, at a ratio of 1∶4 to compare the symptoms related to adverse reactions. In addition, the risk factors of adverse reactions will be analyzed. First, the differences of ADR incidence among the possible influencing factors will be compared separately to find the factors with large differences. Then, synthetic minority oversampling technique and group least absolute shrinkage and selection operator methods will be used to identify factors influencing the occurrence of ADRs, while propensity scoring methods will be used to control for confounding variables.

### Ethical Considerations

This monitoring scheme is implemented after the approval of the Ethics Committee of Xiyuan Hospital of China Academy of Chinese Medical Sciences (ethics batch: 2019XL009-1). Patient data included in the analysis were approved by the institutional review committee of each participating agency. Informed patients in this study were exempt from signing the consent application. In addition, the data will be kept confidential, and the researchers who participate in the data inquiry will need to submit the corresponding application, to ensure that no individual participant or user can be identified in any images in the paper or supplementary materials.

## Results

The first case was included on July 17, 2019. Due to the limited number of eligible patients, missed follow-ups, and the huge clinical burden caused by public health events in 2019, the final case will be enrolled on August 30, 2025.

## Discussion

### Summary

The practice of traditional Chinese medicine in China spans over 2000 years and has consistently garnered high regard for its precise therapeutic efficacy, minimal adverse effects, and cost-effectiveness [33]. As a proprietary Chinese medicine preparation already on the market, PDL had outstanding efficacy in treating URTI [5]. The action mechanism of PDL has been extensively used in clinical practice. However, it is important to acknowledge that there are no completely safe drugs, and every drug administration carries inherent risks. Therefore, the objective of this study was to closely monitor the safety profile of PDL.

The therapeutic safety of PDL is currently being overlooked during the course of disease treatment. Early identification and a better understanding of its adverse reactions will enhance the drug safety profile of PDL and mitigate the therapeutic costs associated with such reactions.

This study is a continuous registry, hospital-intensive monitoring study. Hospital-intensive monitoring allows the evaluation of efficacy and safety in diverse populations under real-world conditions, including sensitive populations that may not be included in premarketing clinical trials, such as pregnant women, ethnic minority groups, older adult patients, children, patients with multiple comorbidities, and those taking multiple medications. The limitation of centralized monitoring in registry hospitals is that it may underestimate the impact of ADRs, as health care professionals may prioritize serious adverse reactions and overlook general adverse reactions. However, this study will implement a 3-level, quality-control approach, using a professional electronic data management system to mitigate missing and misreported data occurrences and ensure the validity and authenticity of the collected information [19].

There are numerous pathological conditions that contribute to drug-induced acute kidney injury, among which the nephrotoxicity of drugs stands as a preventable factor. Enhancing our comprehension of drug safety aids in mitigating the risk associated with drug-induced acute kidney injury. Furthermore, apart from the inherent nephrotoxicity of drugs themselves, administering potentially nephrotoxic medications in high doses or over prolonged treatment durations amplifies the likelihood of renal damage. Additionally, concomitant administration of multiple drugs can synergistically induce nephrotoxicity and consequently lead to kidney injury. Therefore, studying the kidney injury factors of PDL forms the basis for preventing adverse reactions. The Chinese medicine preparation PDL can also induce hepatotoxicity, leading to drug-induced liver damage, which may occur at both therapeutic and overdose doses. This can be attributed to either direct intrinsic hepatotoxicity of the drug or specific (unpredictable) liver toxicity [34], which needs to be further observed and explored.

The monitoring and evaluation of the safety of proprietary Chinese medicine preparations, along with the acquisition of authentic drug safety information, can complement drug instructions, promptly grasp the latest research advancements in related medications, guide their clinical application, and enhance the safety and efficacy of their clinical use.

### Conclusions

This study will obtain the safety results of PDL in real-world, clinical applications. Furthermore, monitoring and evaluating the safety of proprietary Chinese medicine preparation is instrumental in providing further clinical guidance on drug use.

## Abbreviations

ADR: adverse drug reaction
AE: adverse event
ALT: alanine transaminase
CRF: case report form
PDL: Pudilan Xiaoyan oral liquid
SPIRIT: Standard Protocol Items: Recommendations for Interventional Trials
ULN: upper limit of normal
URTI: upper respiratory tract infection
WHO: World Health Organization

## Data Availability

Since this is a protocol, data are not yet available, and information not included in this paper will be provided by the corresponding author upon completion of the trial.
